# Supplementary material for: The association between cadmium exposure and lung cancer risk: A protocol for systematic review and meta-analysis
Source: PLoS One. 2025 Aug 22;20(8):e0329660. doi: 10.1371/journal.pone.0329660 (PMC12373195; doi:10.1371/journal.pone.0329660)
Supplement: S2 File — (DOCX) [file pone.0329660.s002.docx]

**PubMed**

#1 "cadmium"[MeSH Terms] OR "cadmium"[All Fields]

#2 "lung neoplasms"[MeSH Terms] OR ("lung"[All Fields] AND "neoplasms"[All Fields]) OR "lung neoplasms"[All Fields] OR ("lung"[All Fields] AND "cancer"[All Fields]) OR "lung cancer"[All Fields]

#3 #1 AND #2

#4 #3 AND ("humans"[MeSH Terms])

Year of publication can be manually modified from inception to 2024

**WOS**

#1 TS=(Cadmium) OR TS=(Cd)

#2 TS=(lung neoplasms) OR TS=(lung cancer)

#3 = #1 AND #2

In Web of Science, human studies can be only manually screened instead of setting filters.

Year of publication is manually modified from inception to 2024

**Embase**#1 'cadmium':ti,ab,kw OR 'Cd':ti,ab,kw

#2 'lung cancer'/exp OR 'lung neoplas*':ti,ab,kw

#3 #1 AND #2

#4 #3 AND [humans]/lim AND [1900-2024]/py

**Medline (via Ovid)**

1. exp Cadmium/

2. cadmium.ti,ab.

3. 1 or 2

4. exp Lung Neoplasms/

5. (lung cancer or lung neoplas* or pulmonary carcinoma* or pulmonary cancer*).ti,ab.

6. 4 or 5

7. 3 and 6

8. limit 7 to (yr="1946 - 2024" and humans)

**Cochrane Library (CENTRAL)**

#1 MeSH descriptor: [Cadmium] explode all trees

#2 cadmium:ti,ab,kw

#3 #1 or #2

#4 MeSH descriptor: [Lung Neoplasms] explode all trees

#5 (lung cancer OR lung neoplasm* OR pulmonary cancer* OR pulmonary carcinoma*):ti,ab,kw

#6 #4 or #5

#7 #3 and #6

#8 Publication Year from 1946 to 2024

In the Cochrane Library, human studies can be only manually screened instead of setting filters.
