# Supplementary material for: The association between cadmium exposure and lung cancer risk: A protocol for systematic review and meta-analysis
Source: PLoS One. 2025 Aug 22;20(8):e0329660. doi: 10.1371/journal.pone.0329660 (PMC12373195; doi:10.1371/journal.pone.0329660)
Supplement: Attachment — Submitted filename: Response to Reviewers.pdf [file pone.0329660.s007.pdf]

Response to Reviewers

PONE-D-25-15012R1

The Association Between Cadmium Exposure and Lung Cancer Risk: A Protocol for Systematic Review and Meta-Analysis

Reviewers' comments:

Reviewer's Responses to Questions

Comments to the Author

1. Does the manuscript provide a valid rationale for the proposed study, with clearly identified and justified research questions?

The research question outlined is expected to address a valid academic problem or topic and contribute to the base of knowledge in the field.

Reviewer #1: Yes

Reviewer #3: Yes

---

2. Is the protocol technically sound and planned in a manner that will lead to a meaningful outcome and allow testing the stated hypotheses?

The manuscript should describe the methods in sufficient detail to prevent undisclosed flexibility in the experimental procedure or analysis pipeline, including sufficient outcome-neutral conditions (e.g. necessary controls, absence of floor or ceiling effects) to test the proposed hypotheses and a statistical power analysis where applicable. As there may be aspects of the methodology and analysis which can only be refined once the work is undertaken, authors should outline potential assumptions and explicitly describe what aspects of the proposed analyses, if any, are exploratory.

Reviewer #1: Yes

Reviewer #3: Partly

Response: This was exactly the same as the first-round comment. We have addressed issues in a point-to-point approach in our last reply. The manuscript was also revised accordingly.

---

3. Is the methodology feasible and described in sufficient detail to allow the work to be replicable?

Descriptions of methods and materials in the protocol should be reported in sufficient detail for another researcher to reproduce all experiments and analyses. The protocol should describe the appropriate controls, sample size calculations, and replication needed to ensure that the data are robust and reproducible.

Reviewer #1: Yes

Reviewer #3: No

Response: This was exactly the same as the first-round comment. We have addressed issues in a point-to-point approach in our last reply. The manuscript was also revised accordingly.

---

4. Have the authors described where all data underlying the findings will be made available when the study is complete?

The PLOS Data policy requires authors to make all data underlying the findings described in their manuscript fully available without restriction, with rare exception, at the time of publication. The data should be provided as part of the manuscript or its supporting information, or deposited to a public repository. For example, in addition to summary statistics, the data points behind means, medians and variance measures should be available. If there are restrictions on publicly sharing data—e.g. participant privacy or use of data from a third party—those must be specified.

Reviewer #1: Yes

Reviewer #3: Yes

---

5. Is the manuscript presented in an intelligible fashion and written in standard English?

PLOS ONE does not copyedit accepted manuscripts, so the language in submitted articles must be clear, correct, and unambiguous. Any typographical or grammatical errors should be corrected at revision, so please note any specific errors here.

Reviewer #1: Yes

Reviewer #3: Yes

---

6. Review Comments to the Author

Reviewer #1: The authors addressed all my comments and greatly improved what was a good manuscript in the first place. I have no further comments.

Response: Thank you for your kind effort to improve this work.

Reviewer #3: Dear Authors. I appreciate that you have addressed each of my comments and suggestions. However, there are still two areas for improvement:

- The PRISMA-P statement provides recommendations for adequate reporting of a systematic review protocol. It is often mistaken for a guide for developing a protocol, which is incorrect. For example, the statistical methods you have chosen to perform meta-analyses appear in manuals such as the Cochrane Handbook or the JBI manual. These documents correspond to methodological guides to orient the elaboration of protocols. Please correct this.

Response: Thank you for your continued guidance and for acknowledging our attempts to address your initial feedback. We sincerely appreciate your expertise in strengthening the methodological rigor of our protocol. Regarding your concern about the PRISMA-P statement, we now understand that it only serves as a checklist to report the protocol but should not be referred to as a guide to develop the protocol. To correct this, we have revised the manuscript to clarify that the PRISMA-P 2015 statement serves exclusively as a reporting framework, ensuring transparent documentation of our protocol.

The statistical methodology (including selection of random/fixed-effects models, heterogeneity assessment, and sensitivity analyses) is based on established methodological guides: the Cochrane Handbook for Systematic Reviews and the JBI manual. These sources have been cited in the Methods section and added to the reference.

- The information describing your search strategy is still insufficient to be reproducible. The protocol of a systematic review is the point at which aspects such as these are explained in detail. Please revisit the PRISMA-S statement, and report aspects such as: limits and filters used in your strategy (e.g., language and publication status), other search resources (e.g., gray literature, hand searching, searching clinical trial registries), and process how the search strategy was constructed. Be detailed.

Response: Thank you for your kind guidance in improving the transparency and reproducibility of our search methodology. We fully agree that a systematic review protocol must enable exact replication of the search process. We have now comprehensively revised our "Search Strategy" in our manuscript to align with PRISMA-S reporting standards, incorporating the following details:

1) Language: We will not restrict language to include as many studies as possible.

2) Publication Status: Only published articles will be screened.

3) Time Period: Searches will cover all years up to December 31, 2024.

4) Other Filters: Human studies filter was one of our concerns, and we have added human as a filter in the three databases: PubMed, Embase, Medline. Simultaneously, we will manually search WOS and Cochrane library. Detailed information can be found in the revised **S2 File Search strategy**.

5) Grey Literature and Supplementary Search Resources: We will not search for ongoing clinical trials, nor will we search for thesis databases, and we have clarified this in the revised manuscript.

6) Hand-Searching strategy: We only conduct hand-search of references from all included studies and previous systematic review and meta-analyses.

We believe these revisions ensure full compliance with PRISMA-S Items. We also added an additional **S3 File PRISMA-S Checklist** to improve the transparency and reproducibility of the search strategy.

Lastly, we appreciate your guidance in elevating the reproducibility of our protocol. Your insights have been invaluable in refining our protocol’s clarity. We hope these revisions adequately address your concerns and thank you once again for your meticulous review.
