# Supplementary material for: The association between cadmium exposure and lung cancer risk: A protocol for systematic review and meta-analysis
Source: PLoS One. 2025 Aug 22;20(8):e0329660. doi: 10.1371/journal.pone.0329660 (PMC12373195; doi:10.1371/journal.pone.0329660)
Supplement: Attachment — Submitted filename: Reviewer Comments - Protocol Cadmium & Lung Cancer.docx [file pone.0329660.s004.docx]

**Reviewer Comments**

| **Line** | **Paragraph/Section** | **Sentence** | **Revise Suggestion** |
| --- | --- | --- | --- |
| 35-36 | Abstract & Methods | “A comprehensive search of PubMed, Embase, Web 36 of Science will identify observational studies published from inception to 2024” | If possible, please add Cochrane and Medline (OVID) as additional databases. This suggestion is optional, since usually what’s already in those three databases also exist in Cochrane and Medline. |
| 38-39 | Abstract & Methods | “Random-effects models will calculate 39 pooled relative risks (RRs) with 95% confidence intervals (CIs).” | Since it is still protocol, we don’t know whether the heterogeneity will be high (>50%) or moderate to low (<50%). Hence, the author can state something like: *Random-effects model is for high-heterogeneity results, while fixed-effects model is for low-heterogeneity results.* If the author/s are certain the heterogeneity of these types of studies will always be high, then no need to edit. |
| 59-60 | Keywords | Keywords: Cadmium, Heavy metal, Occupational exposure, Lung cancer, 60 Systematic review, Meta-analysis, Protocol | Keywords usually are five (maximum). |
| 63-71 | Introduction | It is primarily associated with lung cancer, as well as 71 prostate, breast, and kidney cancers.[5] | Please add additional reference for prevalence of lung cancer in the world’s population in certain countries, or in Asia. |
| 73-74 | Introduction | Lung cancer (LC) is the most common cancers and the leading cause of death worldwide. | I think this belongs in Paragraph 1. The first paragraph should introduce the phenomenon, and it is essential to close it with prevalence data from epidemiology study or national data statistics. In addition, the opening sentence and the second sentence cite the same source, so it is best to separate them into two paragraphs.  Example from elaborating what author/s wrote:  **Opening of paragraph one:**  Lung cancer (LC) is the most common cancers and the leading cause of death worldwide (6). The prevalence of lung cancer worldwide (or any country and population that become authors’ target) is…..% (source, year). The total death toll caused by lung cancer according to… data is … [source]  **Paragraph two (factor and why cadmium is an important factor)**  It is a multi-factorial disease, causing a substantial economic burden at global, national, and individual levels.[6] While cigarette smoking remains the most important determinant of lung cancer, several other factors, such as environmental factors, e.g., heavy metals exposure could play a substantial role in the etiology and pathogenesis of lung cancer. Heavy metals such as cadmium, are ubiquitous environmental pollutants found in natural environment and occupational settings. Cadmium exposure can lead to its accumulation in lung tissues, where they exert various carcinogenic effects.  **Paragraph three and 4 (short lit review and existing researches, and gap between researches or previous meta-analysis (if any), and why this meta-analysis is necessary):**  Cadmium (Cd), a commonly found heavy metal in industrial society, is contained in soil, rocks, coal, and mineral fertilizers, etc.[1] Its primary route of human exposure is through inhalation and ingestion, posing significant health risks to human owing to its long biological half-life.[1] Epidemiological studies have reported correlation between chronic cadmium exposure and an increased risk of various adverse health outcomes, including cancer incidence and mortality.[2-4] Cadmium is classified as a carcinogen to human by the International Agency for Research on Cancer (IARC). It is primarily associated with lung cancer, as well as prostate, breast, and kidney cancers. [5]  **Research gap paragraph:**  Several meta-analyses have been conducted to evaluate the risk of … to …. A meta-analysis in … (country) reported that cadmium … Another meta-analysis conducted in … (country) summarized that … However, … (Mention the gap in the previous meta-analyses and that the meta-analysis with…. is necessary). Author can re-tailor the discussion part with concise paraphrasing into this part.  Author can also modify their writing: Despite being recognized as cancer carcinogen, and several meta-analyses have been conducted, cadmium’s association with lung cancer risk and mortality are still uncertain. Evidence is lacking regarding cadmium exposure in general population, who is exposed to mild-to-moderate cadmium.[8] (Did the previous meta-analyses reach clear conclusions? If so, what were they? Did they conduct subgroup analyses aligned with the authors’ current interests, as well as sensitivity analyses and publication-bias assessments? If not, these omissions constitute a research gap that the present study will address.)  Last paragraph (your study objective): In this study, we will search all available studies to provide a comprehensive and up-to-date perspective on this topic. Our meta-analysis is designed to fill the previous meta-analyses’ gap by …… (Mention your study strength that will fill the previous meta-analyses’ gap). |
| Line 121 | The record screening is expected to be completed by the end of April. | Eligibility criteria and study selection | Please mention the year |
| Line 157-158 | Statistical analysis | Publication bias will be conducted via funnel plot and further assessed by Egger’s test. | The author can also mention if studies pooled <10, the author do not need to do Egger’s test or further meta-regression, following Cochrane 2023 handbook guideline. |
| Line 165 | Discussion | Three previous meta-analysis reported the correlation between cadmium exposure and lung cancer [2, 167 8, 40]. Chen et al. calculated the risks of general and occupational population separately, marked different gender in the group analysis.[8] | Author/s can mention this in the background too in the research gap paragraph section.  It had better that Chen et al. convert into [source number] as well. For example: One of the aforementioned meta-analyses calculated the risk….   Other citations do not need to be discussed in detail in the Discussion section. The Discussion should mainly cover a brief background of your protocol (maybe 2–3 sentences), the methods you used, the strengths of your study and methodological protocol, and the pivotal challenges in implementing the protocol. All of these points have already been mentioned by the authors, so no major edits (only minor edit) are required. |
| 162-165 | Discussion | This systematic review and meta-analysis will review up-to-date research, and synthesize the evidence on the association between cadmium exposure and lung 164 cancer risk. The results of the systematic review and meta-analysis will provide an insight in both the general and occupational population. | Change this part in the last paragraph of discussion after pivotal challenges/possible limitation, or in the conclusion. |
| 183-185 | Discussion | However, there may be some limitations. First, the between study heterogeneity may be significant, affecting the reliability of the synthesized evidence. | Add additional sentence that: to addresses the pivotal challenges usually occurring in meta-analysis, such as high heterogeneity and publication bias (or the author can choose one of them), author/s will conduct sensitivity analysis by (choose or include them all): subgroup analysis of possible heterogeneity causes, delete-no delete high concern studies to assess fluctuations of I^2^, and funnel plot to see the symmetry of pooled studies, and, if more than ten studies are included, we will follow the Cochrane Handbook (2023) by applying Egger’s test for ….. and….. assessment. If less than 10 studies, we will use qualitative publication bias assessment and Revman’s funnel plot. |
| Figure | Figure 1 | Reports excluded Overlappe | Typo, please revise. |
| References | References | Reference | Please add doi and Pubmed ID or URL for each reference. |
|  |  |  |  |
